# Era of bast fibers-based polymer composites for replacement of man-made fibers

**DOI:** 10.1016/j.heliyon.2024.e29761

**Published:** 2024-04-17

**Authors:** Caroliny M. Santos, Thiago F. Santos, Marcos S. Aquino, Sanjay Mavinkere Rangappa, Suchart Siengchin, Indran Suyambulingam

**Affiliations:** a Textiles Technologies Study Group (GETTEX), Laboratory of Knitting, Department of Textile Engineering (DET), Federal University of Rio Grande do Norte (UFRN), Natal, Rio Grande do Norte, Brazil; b Ecobrasil Industry and Export of Sisal Eireli (ECOBRASIL), Sisaltec Sisal Fiber Industry (SISALTEC), Rodovia Br 101 Norte 10500, Zip code: 59115-00, Natal, Rio Grande do Norte, Brazil; c Natural Composites Research Group Lab, Department of Materials and Production Engineering, The Sirindhorn International Thai-German School of Engineering (TGGS), King Mongkut's University of Technology North Bangkok (KMUTNB), Bangkok, Thailand

**Keywords:** Mechanical properties, Extraction process, Chemical compositions, Technical properties, Industrial applications, Green-composites, Bio-composites

## Abstract

Bast fibers are defined as those obtained from the outer cell layers of the bast of various plant families. They are finding use in textile applications and are widely used as reinforcements for green composites, as bast fibers are perceived as “sustainable”. There is a growing demand for bast fibers across the world due to their renewable and biodegradable nature. The bast fibers are mainly composed of cellulose, which potentially considers the growing techniques, harvesting and extraction processes of bast fibers most used to produce fibers with appropriate quality to apply in the daily lives of modern men and women in contemporary society. This review paper looks at many aspects of natural fibers, with a focus on plant bast fibers, including their impact on prehistoric and historical society. This review shows that bast fibers are competitive compared to man-made fibers in many applications, but variability in mechanical properties and low tenacity may limit their use in high-strengthh composites and extend to, particularly in aerospace, automotive, packaging, building industries, insulation, E-composites (Eco composites), geotextiles and many other applications are currently being explored. Considering, important characteristics of bast fibers include physical, mechanical, and chemical properties. This makes bast fibers one of the most important classes of plant fibers to use as reinforcing agents in thermosetting/thermoplastic polymer matrices. And the effect of bast fibers as reinforcement in the properties of ECO-composites, GREEN-composites, BIO-composites, lightweight composites. Bast fibers play an important role in sustainability, the preservation of the health of the environment, the well-being of the next generation, and even the daily lives of men and women in the contemporary world.

## 1 Background on natural fibers in composites

**Fig. 1:**
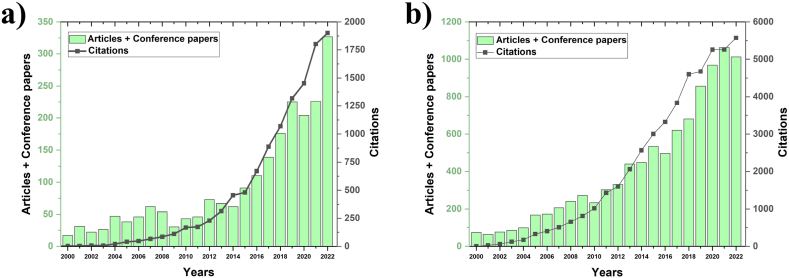
Articles and conference papers published annually from 2000 to 2022 in hybrid and polymeric composites based on a) man-made fibers, and b) natural fibers.

From 2000 to 2022, the number of published articles in the field of hybrid and polymeric composites experienced steady growth as shown in Fig. 1. However, the most significant upsurge occurred after 2010 for natural fibers (Figs. 1a) and 2014 for man-made fibers (Fig. 1b). The adoption of these materials in various industries, driven by environmental concerns and technological advancements, has fueled this exponential increase in research output. The number of citations for articles related to hybrid and polymeric composites also exhibited exponential growth. A notable acceleration was observed from 2010 for natural fibers and 2014 for man-made fibers. Several factors contributed to this surge as breakthroughs in material science and manufacturing technologies have led to the development of more advanced and applicable hybrid composites. This has attracted significant attention from researchers and practitioners alike. With a growing emphasis on sustainable practices, the use of natural fibers gained popularity as an eco-friendly alternative. This shift in focus drew attention to studies exploring the properties and applications of these materials. Increased collaboration between academia and industry has driven the practical application of composite materials, further stimulating research interest and citation rates. The year 2022 witnessed a sharp increase in citations, indicating a pinnacle of interest in hybrid and polymeric composites. This surge was attributed to the successful commercialization of products based on these composites, resulting from earlier research, garnered attention from both academics and industry professionals. The world's increasing focus on sustainability, coupled with a growing demand for high-performance materials, prompted a renewed interest in composite research. However, the evolution of research articles and citations in hybrid and polymeric composites involving synthetic and natural fibers from 2000 to 2022 reflects a dynamic and growing field. The exponential growth in publications and citations is fueled by technological advancements, environmental considerations, and increased collaboration between academia and industry. The sharp increase in citations in 2022 signifies a culmination of these factors, marking a pivotal point in the trajectory of research in this field.

### 1.1 Towards a green world

**Fig. 2:**
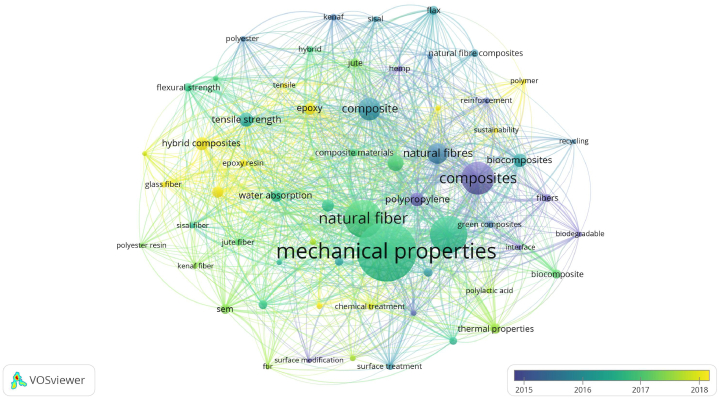
a) keywords used in publications, and b) 62 occurrences of keywords on man-made fibers, and natural fibers used in hybrid and polymeric composites from 2000 to 2022.

The occurrence of keywords in scholarly publications offers valuable insights into prevalent themes and focal points within a specific research domain. Examining the reasons behind the notable frequencies of specified keywords in publications related to natural fibers, mechanical properties, reinforcement, and composite materials from 2000 to 2022, as illustrated in Fig. 2, reveals compelling trends. The term natural fibers is prominent with 5505 occurrences, reflecting a heightened interest in sustainable materials. The escalating emphasis on sustainability and environmental awareness has driven a surge in research exploring the properties and applications of natural fibers as viable alternatives to conventional synthetic materials. Keywords related to mechanical properties appear 3166 times, indicating a keen focus on material performance. A comprehensive understanding of the mechanical properties of composite materials is pivotal for practical applications. Researchers strive to enhance the strength, durability, and overall performance of materials, hence elevating the significance of the keyword mechanical properties. The keyword reinforcement, occurring 3101 times, is integral to the development of composite materials. Researchers concentrate on fortifying materials with fibers to augment their mechanical and structural properties, contributing to the frequent appearance of keyword reinforcement. Fibers, noted 2740 times, is a broad and generic term encompassing both natural and man-made fibers. Its frequent occurrence suggests a thorough exploration of various fiber types and their applications in composite materials. Tensile strength, found 2629 times, is considered a critical mechanical characteristic measuring a material's ability to withstand axial stress. Research consistently emphasizes enhancing tensile strength in composite materials, driving the prevalence of this keyword. The term composites occurs 2313 times, encapsulating the overarching theme of combining different materials to create a superior whole. Researchers investigate a diverse array of composite materials, including those incorporating natural fibers, leading to the high frequency of the keyword composites. Similarly, composite materials appears 2090 times, reflecting the inclusive nature of research in the field. This keyword encompasses studies on the development, characterization, and application of various composite materials. Fiber-reinforced plastics, identified 1794 times, signifies a specific composite type. The occurrence of this keyword suggests a research focus on composites where synthetic or natural fibers serve as reinforcing components within a plastic matrix, indicating the significance of this subtype in various applications. The keyword scanning electron microscopy, appearing 1727 times, is a crucial characterization technique. Its high frequency underscores the substantial emphasis on detailed material characterization in publications, particularly through scanning electron microscopy (SEM). Reinforced plastics, found 1605 times, is a broad term describing materials where a reinforcing agent, often fibers, is incorporated into a plastic matrix. Its frequency highlights the diverse nature of research within the broader category of reinforced materials. In conclusion, the prevalence of these keywords reflects the multidimensional nature of research in hybrid and polymeric composites, highlighting a collective focus on sustainable materials, mechanical properties, reinforcement strategies, and advanced characterization techniques.

### 1.2 The resurgence of bast fibers: revolutionizing industries with sustainable composites

Bast fibers, once marginalized since the advent of the metal age and diminishing significantly with the rise of man-made fibers in modern times, are experiencing a resurgence in technical applications. They are proving to be a sustainable alternative, gradually replacing plastics and contributing to planetary health. Despite their historical fluctuations, bast fibers have demonstrated considerable potential in contemporary society. Their sustainable and renewable characteristics position them as key players in what could be aptly termed the “age of bast fibers” [1]. In the Middle Ages, bast fibers served as the foundation of the economy through innovative devices like fishing nets. However, in the 19th century, they faced another decline, exacerbated by the loss of profitability and commercial viability compared to emerging man-made fibers like nylon and polyester. This led to a drastic reduction in the cultivation of bast fibers, with some countries seeing their disappearance. Seemingly on the brink of obsolescence, bast fibers have resurfaced today as crucial components in the creation of cutting-edge materials known as “composites,” breathing new life into their role [2].

**Fig. 3:**
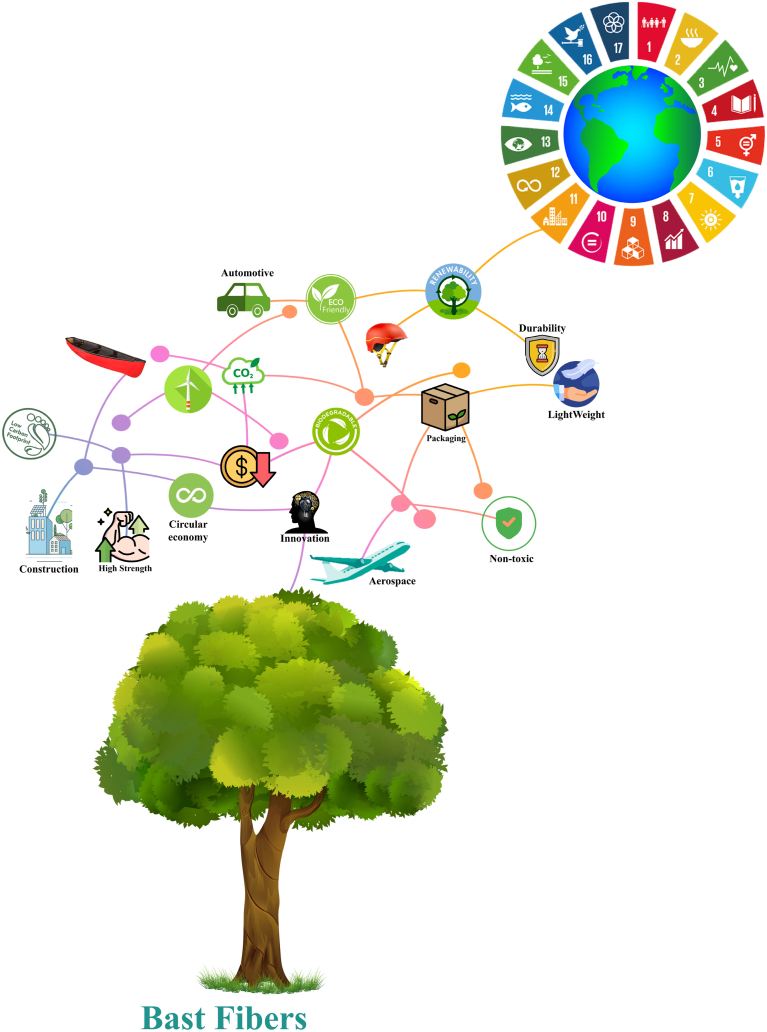
Applicability of bast fibers currently discovered.

In recent decades, the renewed interest in bast fibers stems from their commendable mechanical properties, renewable potential, contribution to circular economy practices, widespread availability, and cost-effectiveness compared to synthetic and metallic alternatives [3]. Beyond their technical advantages, the application of bast fibers also offers environmental benefits, such as carbon dioxide absorption and lower energy consumption during production compared to glass and man-made fibers [[4], [5], [6]]. The durability of bast fiber composites makes them valuable in various sectors, including food packaging, aerospace, construction, automotive, and other emerging fields, contributing to economic, creative, and sustainable growth. The versatility of bast fibers has spurred researchers to explore new varieties of plant fibers, leading to a growing demand for products based on renewable materials [7]. Looking ahead, the focus on developing and innovating new products incorporating bast fibers has become a central theme for engineers, designers, companies, markets, and consumers alike. Far from being mere components, bast fibers have evolved into the driving force behind global market consumption, fueled by significant political incentives. The anticipation is for plant-based fibers to play an increasingly substantial role in diverse industrial sectors worldwide [1,8]. Consequently, just as metals like iron, bronze, and copper once marked innovative raw materials, the present marks the era of bast fibers [7,8]. Bast fibers, derived from the inner bark of certain plants, have been used for various applications throughout history due to their strength, durability, and versatility. Some common bast fibers include flax, hemp, jute, and ramie. In recent times, these fibers have found applications in diverse industries, including packaging, aerospace, building, and automotive. Bast fibers are increasingly being used in the packaging industry as an environmentally friendly alternative to traditional materials. The fibers' high tensile strength and resistance to tearing make them suitable for manufacturing durable and robust packaging materials. Products such as bags, sacks, and nets made from bast fibers are not only strong but also biodegradable, reducing environmental impact. The aerospace industry values bast fibers for their lightweight yet strong properties. These fibers are used in the manufacturing of composite materials that find applications in aircraft components. The lightweight nature of bast fiber composites contributes to fuel efficiency, making them an attractive option for aerospace engineers. Additionally, bast fibers offer good thermal insulation properties, further enhancing their suitability for aerospace applications. In the construction and building industry, bast fibers are utilized for reinforcement in composites and as raw materials for manufacturing various construction components. These fibers are often incorporated into concrete to enhance its tensile strength and reduce cracking. Bast fibers can also be used in the production of insulation materials, providing both thermal and acoustic insulation in buildings. Their eco-friendly nature aligns with the growing emphasis on sustainable and green construction practices. The use of bast fibers in the automotive sector enhances the production of interior parts like door panels, seat backs, and dashboards. These fibers, incorporated into composite materials, strike an excellent balance between strength and lightness. This lightweight feature boosts fuel efficiency and the overall performance of vehicles. Additionally, the versatility of bast fibers allows them to be molded into diverse shapes, offering automotive manufacturers considerable design flexibility. Beyond the automotive realm, bast fibers have found valuable applications in the packaging, aerospace, and construction industries. Their remarkable blend of durability, lightness, and environmental friendliness makes them a sustainable choice across various manufacturing domains. As the global emphasis on environmental sustainability intensifies, the demand for bast fibers is expected to surge, driving towards a greener and more eco-conscious future (see Fig. 3).

## 2 Classification of plant fibers to reinforce composites

**Fig. 4:**
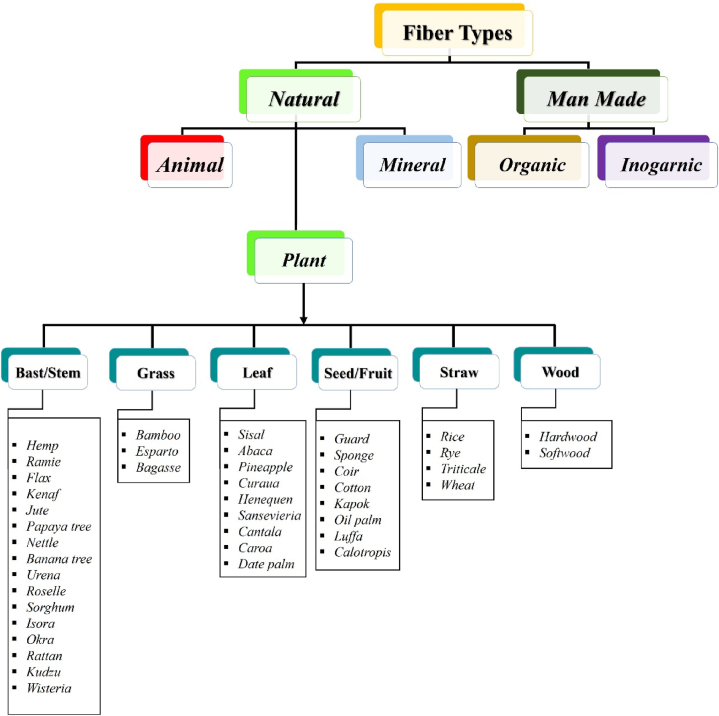
Classification of the main plant fibers currently discovered.

For centuries, bast fibers have been derived from plant sources, playing a pivotal role in the commercial production of diverse fabrics, paper, and engineering materials [9]. Fig. 4 illustrates the categorization of fibers into natural and man-made, with plant fibers further classified into bast/stem, grass, leaf, seed/fruit, straw, and wood fibers. Central to all plant fibers is cellulose, making them inherently more biodegradable and easier to recycle. Generally, plant fibers boast commendable strength and stiffness [10]. Notably, bast fibers emerge as the most suitable and cost-effective choice for the production of bioproducts [11].

In the contemporary era, there has been a notable surge in the demand for knowledge concerning plant fibers and composites reinforced by bast fibers, particularly in major global economic powers. This trend underscores the escalating interest in plant fibers, predominantly those of bast/stem origin (bast lignocellulosic fibers). While numerous plant bast species contribute to the bast fiber pool, only a select few have garnered commercial prominence. Nevertheless, it's noteworthy that approximately 40.3 % of the world's recognized bast fibers originate from plant bast, as highlighted in Fig. 4 [12].

## 3 Extraction process

The choice of extraction method is critical, determining the characteristics and properties of the extracted bast fibers and, consequently, the composites manufactured from them [17,18]. These extraction methods are selected based on properties such as fiber length, duration, and the advantages and disadvantages inherent in their applicability. Bast fibers, known for their sustainability, are highly regarded over synthetic counterparts [19,20]. Their economical acquisition and environmental advantages make them superior in various applications [21,22]. Presently, bast fibers are extensively explored for use in green composites, aiming to create ecological, sustainable materials with lightweight performance for diverse applications [[23], [24], [25]]. Despite typically having a shorter usage lifespan, bast fibers are often fully or partially recyclable or biodegradable [26,27].

### 3.1 Mechanical process (decortication)

Bast fibers can be extracted from plant stalks through a mechanical process known as decortication. This method is commonly employed for leaf fibers like sisal, which do not undergo the typical retting process associated with bast fibers [69]. In the decortication field, plant stalks are carefully cut or prepared before being fed into a decorticator machine. Prior cleansing of the stalks is crucial to minimize potential damage to the bast fibers during the extraction process. Within the decortication process, non-fibrous components undergo crushing and beating between rollers equipped with blunt knives. The resulting pulp, or mucilage, is mechanically scraped away from the bast fibers [70]. Throughout this process, the entire organic structure of plant basts is mechanically fragmented into smaller pieces (shives or mucilage), necessitating complete breakdown to obtain the desired bast fiber bundles [71]. The extraction of bast fiber for technical applications involves the destruction of the stem, stalk, or bast, or other plant parts containing fiber tissues, such as the vegetable fibers obtained from leaves [72]. Post-decortication, the extracted fibers undergo a washing phase, typically through immersion in water tanks to remove mucilage, pectin, and other plant residues. Subsequently, the fibers are dried using mechanical means (hot air) or natural methods (sun drying). Following drying, the fibers undergo testing and quantification of physical, mechanical, chemical, and thermal properties. The results guide their application in the development of innovative lightweight materials. This extraction process is crucial for obtaining the necessary quantity and quality of bast fibers, considering characteristics such as fineness, length-to-diameter ratio (L/D), density, and mechanical properties, especially the modulus of elasticity to density ratio (MOE/ρ).

### 3.2 Retting process in water (microbiological)

In the retting process, bast fibers are separated from plant stalks through a microbiological method that involves water removal [73]. This method relies on microorganisms breaking the chemical bonds, particularly those of pectin, hemicellulose, and lignin, that bind the stalk components together. This action allows for the extraction of bast fibers from the plant, separating them from the woody space, epidermis, and other components [74,75]. In water retting, the plant stalks are cut or pulled from the soil and left to decompose in rivers, tanks, and barrels in the field, where microbial activity takes place. This traditional water retting method involves immersing the stalks in water [76]. Among various fiber extraction methods, water retting stands out as the oldest and most commonly used for stalks. Bacteria and fungi, activated during soaking, break down both the soft and hard tissues of the stalks, leaving the bast fibers (rich in cellulose) intact. Subsequent washing removes the softened tissue, resulting in bast fibers with the removal of the outer cuticle and epidermal layer, leaving the central pith in the fibers [77,78]. The bast fibers consist of vascular tissue, comprising xylem vessels and sclerenchyma fibers [79,80]. In the retting methodology, mechanical, thermal, chemical, and physical properties vary significantly, influenced by the fiber's chemical composition, biological family, species, and environmental conditions [81,82]. Although slower and less environmentally friendly than mechanical extraction, water retting produces lower-purity bast fibers [83,84]. This method yields less uniform, acceptable quality fibers, and is more expensive, generating nitrogen-rich wastewater requiring treatment before discharge. Consequently, the production of solid and liquid waste raises environmental concerns and limits the range of applications for these extracted fibers [85].

### 3.3 Boiling process

In the pursuit of innovative industrial applications, bast fibers need to be initially separated from the non-fibrous components of the stalk [86]. To achieve this, plants undergo a cooking process known as boiling immediately after harvesting and cleaning the stalks. Boiling is a physical-chemical procedure where time, pressure, and temperature work together to break the chemical bonds, particularly those rich in cellulose, that bind the non-fibrous components of the stalk, such as pectin, hemicellulose, and lignin. This process allows for the extraction of bast fibers [78].

Presently, hot water boiling stands out as the most widely accepted method for extracting bast fibers. In this method, bunches of stalks are immersed in hot water, resulting in the removal of the outer cuticle and epidermal layer, leaving the central pith in the bast fibers [87]. This boiling extraction process is faster and more environmentally friendly compared to water retting, making it a significant step in the manufacture of bast fibers. It plays a crucial role in influencing fiber quality and, consequently, their application field [87]. Boiling extraction is known for producing more uniform and higher-quality fibers, characterized by a lighter color, as the hot water effectively removes most of the dirt and non-fibrous components [88,89]. After extraction, the bast fibers undergo testing and quantification of physical, mechanical, chemical, and thermal properties, directing their application towards the development of innovative lightweight materials and technical uses. This extraction process, along with subsequent technical applications, follows retting, mechanical, and boiling procedures, ensuring the complete removal of nonfibrous materials, as outlined in Table 1, which compares the main extraction processes of bast fibers.

**Table 1:** Comparison between main extraction process of bast fibers.

Characteristics	Extraction by boiling	Extraction by retting	Extraction by mechanical methods
**Favorable**	Requires medium-low cost to use in industrial scale and produce bast fibers of high quality. Cleaner bast fibers because virtually all the mucilage is removed. It exhibits better mechanical properties, and higher fiber yield, due to less waste during extraction when compared to other extraction processes.	Most used and simple extraction process due to its breadth of worldwide knowledge and production bast fibers of acceptable quality and uniformity. Most accessible extraction method water (plant bast needs submersed in rivers or tanks). It can be applied to several families of plants to extract their fibers mainly for hard fibers.	It currently produces large quantities of short fibers in a short time and has several models of machines developed for industrial scale and in a variety of bast fibers. Faster and more environmentally friendly compared to Extraction by retting.
**Unfavorable**	Depending on the bast the process is less effective. This process is most efficient when boilling is done under pressure. After the boilling process, it is necessary to wait a few hours for the basts to cool down to extract the bast fibers. It needs to be developed on an industrial scale (develop a pilot process).	Requires medium-high cost to use on industrial scale, high maintenance, and water treatment. It poses risks to human and environmental health. Raises environmental concerns, and inferior fibers quality compared to bast fibers obtained through boiling and mechanical process. Slower and less environmentally friendly compared to Extraction by mechanical.	Extraction process is commonly used industrially and complex due to its commercial scope and more expensive. Produces large volumes of solid waste in the textile fiber extraction industry. Risk of minor limb amputations in machine operators. High consumption of energy or fossil fuels during the production of the bast fibers. The method is also unsuitable for extracting some fibers from the bast, such as papaya fiber, due to the difficulty in removing the large amount of mucilage combined with the high stiffness of the bast, which can lead to the fibers breaking during the process. In this case, the most appropriate method would be boiling extraction.
**Description**	Bast fibers are boiled water in a hermetically sealed container (extractor) to be subjected to temperature and pressure or not. During a period of a time determined for better separation of fibers, this condition it must be sufficient for softening of non-fibrous material. After, the drying of bast fibers are done under natural environmental conditions (under wind and sun).	Bast fibers are submerged in water in rivers, barrels, and tanks to be subjected to the action or action of a microorganism (bacteria and/or fungi). During a determined period of time for better separation of the bast fibers, this condition must be enough for the microorganisms to attack the non-fibrous materials and checked periodically to guarantee the quality and properties of the fibers.	Bast fibers are inserted into a cylinder of blades or angle brackets that rotate at high speed (hammer mill or/and decorator), in this phase the fiber is separated from the mucilage. The feeding of the bast can be manual or automatic with the aid of a feeding tray. In a few seconds, the wet bast fiber is obtained and after, the drying process of bast fibers are done under natural environmental conditions (under wind and sun).
**Duration**	It depends on the initial preparation of the bast (cutting and cleaning), and the type and time of the drying process (time consuming), however, the fibers are extracted few hours (on average lasts about 1–5 Hours)	It depends on the initial preparation of the bast (cutting and cleaning) and mainly on the amount of non-fibrous material present in the bast and the time of the drying process (moderate), however, the fibers are extracted in a few days (it lasts on average about 7–18 days).	It depends on the initial preparation of the bast (cutting and cleaning), and the type and time of the drying process (time consuming), however, the fibers are extracted practically instantly.

## 4 Properties and characteristics of bast fibers

### 4.1 Physical properties

The shift towards environmental sustainability has become a key factor in driving the innovation of eco-friendly composite materials, with references highlighting this trend [90,91]. Bast fibers have been spotlighted as a renewable substitute for synthetic fibers in crafting eco-conscious composite products [11,91]. Recently, multiple sectors have begun replacing synthetic fibers with bast fibers, leveraging their benefits for lighter constructions, cost savings, and energy efficiency [92,93]. In assessing bast fibers' quality, their wettability, density, and production yield stand out as essential metrics. The density of these fibers can differ depending on their specific location in the plant, which directly correlates to their designated function within the plant's stalk [92,94]. This density variation significantly affects the overall weight of the composite material. Table 2 offers a detailed comparison of the key physical characteristics of various recently explored bast fibers, shedding light on their unique properties and potential applications.

**Table 2:** Comparison of the main physical properties between various bast fibers.

Bast fibers	Scientific name	Wettability (Degrees)	Density (g/cm³)	Fiber yield (%)
**Banana tree**	*Musa acuminata*	45.3–102 [95,96]	1.35 [97]	1–2 [98]
**Flax**	*Linum usitatissimum*	91 [99]	1.40 [100]	5–17.0 [101,102]
**Hemp**	*Cannabis sativa*	58 [103]	1.48 [100]	17–40 [104,105]
**Jute**	*Corchorus capsularis*	82 [106]	1.30 [107]	6 [108]
**Kenaf**	*Hibiscus cannabinus*	55–87 [109,110]	0.6–1.50 [111]	5–6 [112]
**Nettle**	*Urtica dioica*	–	0.5–1.0 [113]	11 [114]
**Papaya tree**	*Carica papaya Linn*	78–98 [89,115]	0.65–1.13 [89,115]	2.7–15.3 [89,115]
**Ramie**	*Boehmeria nivea*	75.9 [116]	1.50 [117]	2–5 [118]
**Roselle**	*Hibiscus sabdariffa*	–	1.49 [119]	3.7–4.9 [120]
**Urena lobata**	*Caesarweed*	–	0.88–1.0 [121]	–
**Isora**	*Helicteres isora* L.	–	1.2–1.39 [[122], [123], [124]]	–
**Rattan**	*Calamus manan*	–	0.33–0.6 [124,126]	–
**Sorghum**	*Sorghum bicolor* L.	–	1.5 [127]	–
**Okra**	*Abelmoschus esculentus*	–	1.15–1.45 [128]	4–14.7 [129,130]

### 4.2 Chemical composition

Bast fibers are derived from the inner bark of various plant families, with cellulose being their primary and most abundant component [13]. Comprising cellulose, hemicellulose, lignin, pectin, and waxes, bast fibers undergo traditional extraction processes post-retting, mechanical treatment, and boiling, ensuring the complete removal of nonfibrous materials [14,15]. The sustainable and efficient processing of bast fiber involves mandatory stages such as the preparation and extraction of bast in the plant, fiber extraction, and subsequent optional stages like bleaching, dyeing, softening, printing, and drying, culminating in the production of environmentally friendly materials [16]. Elementary bast fibers primarily consist of cellulose, hemicellulose, and are bound together by pectin, while a woody, pithy inner core runs through the middle of the bast. The hollow space, known as the lumen, aids in nutrient transport within the plant. The length of bast fibers, indicated by the length-to-diameter ratio (L/D), is significantly larger, and their microscopic cross-section reveals a structure from the epidermis surrounding the cortex to the primary fiber bundles located within. The fine structure of bast fibers consists of elongated bundles of fibrils, each exhibiting a dimension relationship similar to the fiber. These fibrils are composed of macromolecules, mainly cellulose (α-cellulose), hemicellulose, lignin, pectin, and wax in lower percentages [[28], [29], [30], [31], [32], [33]]. The data presented in articles, chapters, books, and conference papers reveal substantial variations in the final composition of bast fibers compared to man-made fibers. These deviations arise from numerous uncontrollable factors, including but not limited to the country of origin, climate conditions, and extraction processes [34,35]. The elementary bast fibers consist of multiple microfibrils, forming bundles bound together by hemicelluloses [36]. Lignin serves as the bonding agent on the external structure of the bast fiber bundles. Within these fibers, cellulose macromolecules organize into microfibrils, featuring highly structured (crystalline) regions alternating with less organized (amorphous) regions [37,38].

#### 4.2.1 Cellulose

Cellulose serves as the foundational structure of bast fibers, presenting itself as a linear macromolecule [39,40]. This cellulose framework adopts a three-dimensional (3D) structure, facilitated by the establishment of the maximum conceivable number of hydrogen bonds within the crystalline regions [41,42]. The linear configuration of cellulose molecules imparts a robust inclination to engage in both intramolecular and intermolecular hydrogen bonding, forming connections with themselves and other cellulose or polar molecules [43,44]. Cellulose stands out as the primary and pivotal component in the structure of bast fibers, often referred to as lignocellulosic materials [133]. The composition of compounds within bast fibers, classified as lignocellulosic fibers, encompasses cellulose, hemicellulose, lignin, wax, and pectin [134,135]. Evaluating the properties of bast fibers necessitates consideration of their crystallinity, a crucial parameter [136,137].

Despite containing identical elements, the composition of bast fibers varies, resulting in distinct characteristics when employed as reinforcement in green and sustainable composites [138]. This diversity in composition significantly influences the applicability and performance of the composite, limiting them based on the unique chemical attributes of each bast fiber [139]. Cellulose plays a pivotal role in maintaining the structure, strength, and rigidity of bast fibers, with increased cellulose content contributing to improved alignment of fibrils along the fiber axis, leading to enhanced tensile strength and stiffness [[140], [141], [142]].

#### 4.2.2 Hemicellulose

Following cellulose, the second most prevalent component in the plant cell wall is hemicellulose. Consequently, hemicellulose generally exhibits greater flexibility and solubility compared to cellulose [45,46]. Both hemicellulose and cellulose are interconnected through robust forces like hydrogen bonds and van der Waals forces, making their separation challenging. The chemical structure of hemicellulose involves lower polymerization degrees than cellulose, comprising both homopolysaccharides and heteropolysaccharides (molecules containing more than one sugar or sugar derivative). These substances act as a supporting material within the cell wall structure, akin to cellulose [[47], [48], [49]]. Hemicellulose in bast fibers increases: moisture content, Young's modulus, Specific Modulus (E/ρ) and specific strength while decreasing tensile strength, failure strain, thermal stability, and density [143]. In contrast, the linkage between hemicellulose and lignin involves both covalent and non-covalent bonds. The extensive network of chemical and physical interactions among cell components enhances both the rigidity and flexibility of the plant cell wall, consequently influencing the properties of bast fibers [50,51].

#### 4.2.3 Lignin

The term “Lignin” is derived from the Latin word for “wood,” indicating a high-molecular, three-dimensional (3D) amorphous polymer that binds fibers and vessels within wood and plants [[52], [53], [54]]. Lignin exhibits diverse chemical structures and compositions, resulting in varied properties depending on its source, which can include trees, crops, and various plants [55,56]. Predominantly located in the walls of secondarily thickened cells, lignin imparts rigidity and impermeability to these structures [57]. Due to variations in its subunit composition and intermolecular linkages, lignin lacks a precise and uniform definition [58]. The stiffness of bast fibers is predominantly influenced by the lignin composition, as these 3D, amorphous polymers act as adhesives within the fibers [144]. A higher concentration of lignin enhances the stability of the bast fiber structure but diminishes its strength [71,145].

#### 4.2.4 Wax

These chemical components are crucial elements in the structure of bast fibers, primarily located in the epidermis and the outer section of the bast. They act as barriers, preventing drying and microbial intrusion within the plant bast [49,59]. Furthermore, these components play a pivotal role in determining the flexibility and soft texture of bast fibers, contributing to a reduced friction during the processing of raw bast fibers into various components [[60], [61], [62]]. Waxes, mixtures of long-chain aliphatic hydrocarbons, hinder wetting during processing, potentially compromising engineering properties that enable application [146,147]. Lower wax content fosters hydrogen bonding between cellulose fibril chains, enhancing mechanical strength performance.

#### 4.2.5 Pectin

Pectin is a vital component of the cell walls in fiber basts, representing a structurally and functionally intricate polysaccharide within bast cell walls [63,64]. Comprising a complex mixture of polysaccharides, pectin constitutes approximately one-third of the cell wall's dry substance in higher plants [65]. Within the plant bast, pectin serves as a binder, uniting plant components to ensure cohesion between bast fibers and providing stability to the plant tissue, particularly the epidermis [66]. Consequently, the strength of the plant cell wall hinges on the orientation of these chains, exerting a significant influence on the mechanical properties of bast fibers [67,68]. The crystallinity index, a critical microstructural parameter, influences the mechanical properties of bast fibers. A higher crystallinity index corresponds to increased hardness and density [[148], [149], [150]]. Regarding the diverse opinions on the effect of pectin content on fiber properties, this complexity is likely due to the different types of bast fibers, methods of pectin quantification, and the varied conditions under which these studies are conducted. The literature on this subject is extensive, and studies may use different plant sources, extraction methods, and analytical techniques, leading to a range of findings. However, the consensus appears to be that pectin plays a significant role in determining the mechanical and physical properties of bast fibers, offering pathways for tailored fiber development for specific industrial applications. Examining the chemical composition is imperative for gaining a comprehensive understanding of the nature of bast fibers and their suitability for diverse applications in sectors like automotive, textiles, packaging, construction, and maritime industries [131,132]. The chemical components present in the primary bast fibers are detailed in Table 3.

**Table 3:** Comparison of the main chemical compositions between various bast fibers.

Bast fibers	Cellulose (%)	Hemicellulose (%)	Lignin (%)	Waxes (%)	Pectin (%)	Crystallinity index (%)
**Banana tree**	60-65 [151]	6-19 [151]	5-10 [151]	–	3-5 [151]	39 [2]
**Flax**	71 [2]	18.6–20.6 [2]	2.2 [2]	1.5 [2]	2.3 [2]	86.1 [2]
**Hemp**	55–90 [152]	15–22.4 [152]	4–13 [152]	0.8 [117]	0.9 [117]	79.9 [2]
**Jute**	61–71 [117,153,154]	12–20 [117,153,154]	12–13 [117,153,154]	0.5 [117,154]	0.4 [117,154]	58 [2]
**Kenaf**	72 [117,154]	20.3 [117,154]	9 [117,154]	–	–	67.0–79.8 [155,156]
**Nettle**	78.5–88.5 [114]	0.7–12.3 [114]	2.3–5.9 [114]	–	–	–
**Papaya tree**	50.5–58.7 [157,158]	11.8–29.4 [157,158]	14.3–20.3 [157,158]	0.8 [158]	–	59.6–63.1 [89,115]
**Ramie**	68.6–76.2 [117,153,154]	13.1–16.7 [117,153,154]	0.6–0.7 [117,153,154]	0.3 [117,153,154]	1.9 [117,153,154]	62.9 [2]
**Roselle**	56.3 [119]	11.5 [119]	7.3 [119]	0.5 [119]	–	–
**Urena lobata**	73.2 [153]	10.6 [153]	16.2 [153]	–	–	–
**Isora**	74.8 [124,159]	–	23.0 [124,159]	1.09 [159]	–	–
**Rattan**	73.8 [160]	12.49 [160]	10.15 [160]	–	0.37 [160]	67.03 [160]
**Sorghum**	41.3–46.1 [184]	11.7–15.6 [184]	11.5–13.9 [184]	–	–	–
**Okra**	67.5 [162]	15.4 [162]	7.1 [162]	3.9 [162]	3.4 [162]	–

### 4.3 Mechanical properties

The mechanical properties and overall performance of products crafted from bast fiber composites hinge on various factors, including processing techniques, the intrinsic properties of their individual components (fiber-matrix), and the compatibility and interfacial bonding between the polymer and bast fibers [163]. A notable distinction between bast fiber and man-made fibers lies in the considerable variability in mechanical properties [164,165]. The diversity in the mechanical characteristics of bast fibers can be attributed to factors such as different fiber sources, plant age, fiber maturity, climatic conditions, and extraction techniques [166]. Within the realm of bast fibers, the extracted fibers showcase the most varied and distinct mechanical properties discovered to date [167]. This diversity has spurred efforts to explore new bast fiber reinforcements for potential use in polymeric matrix composites that are environmentally friendly, causing no harm to the environment and decomposing naturally without releasing toxic pollutants into the atmosphere [168,169]. Table 4 provides a comprehensive overview of the main mechanical properties across the spectrum of discovered bast fibers, highlighting their versatility.

**Table 4:** Comparison of the main mechanical properties between various bast fibers.

Bast fibers	Elongation at Break (%)	Tensile strength (MPa)	Young's modulus (GPa)	Specific modulus – E/ρ (GPa)
**Banana tree**	2 [170]	53.7 [171]	27-32 [172]	20–23.7
**Flax**	1.6–2.5 [173]	510–910 [174]	50–70 [175]	34–48 [175]
**Hemp**	1.6 [175]	300–760 [174]	30–60 [174]	20–41 [174]
**Jute**	1.5–1.8 [107]	200–770 [107]	20–55 [107]	2–37 [107]
**Kenaf**	2.7–6.9 [176]	300–1200 [174]	22–60 [174]	17–46 [174]
**Nettle**	1.5–4.3 [177]	598.2–954 [178,179]	19.2–115 [178,179]	13.2–79.3 [180]
**Papaya tree**	1.2–2.3 [89,115]	3.8–12 [89,115]	0.2–2 [89,115]	0.3–3.2 [ [89,115]
**Ramie**	2.0–3.8 [117]	400–938 [117]	44–128 [117]	29–85 [117]
**Roselle**	0.7 [181]	80.2–562 [181]	7.5–18.8 [182,183]	5.5–3.8 [181,182]
**Urena lobata**	2.3–3.8 [121]	214–215 [207]	5–20.5 [121,184]	4–16.3 [184,185]
**Isora**	5.0–6.0 [122,159]	500–600 [122,159]	18-20 [159]	1.3 [122,159]
**Rattan**	2.7–3.2 [125]	42–57.2 [125]	1.2–2.2 [125]	2.8–5.1 [125]
**Sorghum**	1.6–3.2 [161,186,187]	188–326 [161,186,187]	9.8–21.1 [161,186,187]	–
**Okra**	2.5–8.6 [128,188]	234–380 [128,188]	5–13 [128,188]	4.4–9 [128,188]

Bast fiber-reinforced composites represent a green material poised for future engineering applications [189]. In the current landscape, terms such as “green,” “environmentally correct,” and “sustainability” have become pivotal criteria in the creation, development, and design of products for both domestic and industrial use. There is an increasing imperative for research and development (R&D) efforts to be oriented toward the utilization of environmentally friendly and sustainable materials, steering away from fossil-based, non-biodegradable, toxic, and non-renewable resources, as detailed in Table 5. Currently, materials that align with ecological and sustainable principles are actively considered for new product development due to their versatility and the advantageous attributes they bring, contributing environmental, social, and economic benefits over the short, medium, and long term.

**Table 5:** Comparison of technical, economic, processability, availability, and ecological characteristics between bast fibers and man-made fibers.

Characteristics	Bast fibers	Man-made fibers
**Abrasiveness**	Low	High
**Annual global production**	Low	High
**Biodegradability**	Biodegradable	Nonbiodegradable
**CO**_**2**_**neutral**	Yes	No
**Chemical nature**	Lignocellulosic	Petroleum or petrochemicals
**Cost**	Higher than Man-made fibers	Low
**Density**	Low	High than bast fibers
**Distribution**	Wide	Wide
**Economy type**	Circular	Linear
**Elongation at Break (%)**	0.7–8.6	–
**Energy consumption**	Low	High
**Embodied energy to process commercial raw bast fibers (MJ/kg)**	4–15	20–37
**Fiber length**	Discontinuous	Continuous
**Health risk/Hazardous/toxic (upon inhalation)**	No	Yes
**Incentive to agriculture**	Yes	No
**Mechanical properties**	Moderate	High
**Moisture sensitivity**	High	Low
**Recyclability**	Good	Moderate
**Renewability**	Renewable	Nonrenewable
**Renewable source**	Yes (Infinite)	No (Limited)
**Specific modulus – E/ρ (GPa)**	0.3–85	28–171.4
**Tensile strength (MPa)**	3.8–1200	27–4000
**Thermal sensitivity**	High	Low
**Toxicity**	Nontoxic	Toxic
**Young's modulus (GPa)**	0.2–128	0.5–240

Materials reinforced by bast fibers offer industries the capability to generate a substantial volume of green products without compromising the health of our planet. Sustainable composites face significant preconceptual barriers but are emerging as focal points for advanced applications [190], positioning themselves as pivotal components in the journey toward circularity. These sustainable composites represent future prospects to meet market demands across diverse sectors such as automotive, aerospace, construction, household products, electronics, biomedical applications, and packaging industries [191]. Both government and private funding agencies are channeling substantial resources into the development of eco-friendly products [192]. Consequently, sustainable composites have become a focus for researchers, academia, and scientists, leading to innovative ideas for composites reinforced with renewable resources as alternative materials. This review paper aims to highlight the technical characteristics of bast fibers and the potential of new products and markets. It emphasizes the strong potential of these sustainable materials, especially in applications with reduced environmental impact. The paper includes a comparative analysis of technical, economic, processability, availability, and ecological characteristics between bast fibers and man-made fibers. It also underscores the application of bast fibers in developing new products as an alternative to synthetic materials derived from fossil sources [193] as outlined in Table 5.

## 5 Chemical treatment of bast fibers

Like any other natural plant fiber, bast fibers are naturally hydrophilic due to their main components, which include cellulose, hemicellulose, and lignin. These components possess numerous hydroxyl groups, attributing to the fibers' hydrophilic nature and thus their significant moisture sensitivity. Although this high moisture sensitivity stands as the chief barrier to the industrial use of plant-based fibers, these surface functional groups are essential for fiber chemical modification. Such modifications enhance the fibers' polarity, greatly improving their bonding with hydrophobic polymers. Chemical treatments, such as alkali and silane treatments, are commonly employed to increase fiber hydrophobicity, with alkali treatment often serving as a preliminary step to eliminate surface residues, thereby enhancing fibrillation and surface texture.

### 5.1 Alkali treatment

Alkali treatment, specifically known as mercerization, stands as the prevalent method for processing plant fibers [[206], [207], [208]]. This technique primarily focuses on purifying the fiber surface, leading to the emergence of rougher textures. The process typically employs a sodium hydroxide (NaOH) solution with concentrations varying from 5 % to 20 %, and the duration of treatment can span from 1 to 5 h, influenced by the temperature utilized. For instance, ramie fibers have been treated using a 5 % NaOH solution for 4 h at ambient temperature, followed by washing and neutralization with deionized water containing acetic acid to ensure the complete elimination of NaOH [209]. Post-treatment, the fibers are dried under vacuum at 98 °C to prepare them for hybrid applications. This method enables fibrillation by partially removing substances like gums and pectin, which bind the fibrils together. Similarly, Saw et al. applied a 5 % NaOH solution to jute fibers at 30 °C, maintaining a liquor ratio of 15:1 for 2 h, before combining them with bagasse fibers to fabricate hybrid composites [210]. Observations revealed cracks or pits on the fiber surfaces, attributable to the partial removal of fatty substances and wax.

### 5.2 Silane treatment

To enhance the compatibility between ramie fibers and their polymeric hosts, a silane treatment process was executed [209,211]. This process significantly improved the wettability and adhesion of the functionalized ramie fibers within the polymeric matrix, as illustrated by their entrapment within the host material. Such improved interactions facilitated better stress distribution from the polymer to the fibers, thereby boosting the composite's mechanical performance. Conversely, untreated ramie fibers exhibited poor adhesion, leading to fiber pullouts and consequent voids in the matrix [211]. In related studies, NaOH treatments were applied to kenaf and pineapple leaf fibers before silane application, aiming to strengthen fiber-matrix bonds. However, these treatments varied in effectiveness across different fiber types, influencing moisture absorption and mechanical properties, with pineapple leaf fibers showing superior results due to their high cellulose content. Additionally, maleic anhydride grafted polypropylene (MA-g-PP) has been explored as another coupling agent to enhance fiber-polymer interaction. This approach, sometimes combined with alkali treatments, has shown promising improvements in interfacial adhesion, mechanical strengths, and flame resistance. However, secondary modifications, such as applying ammonium polyphosphate for flame retardancy to silane-treated fibers, can compromise the beneficial interactions between fibers and polymer, highlighting the complexity of achieving desired enhancements in composite materials [209].

### 5.3 Microbiological treatment

The application of enzymes is becoming a popular eco-friendly method to improve fiber quality within the composite materials sector [212]. The primary aim of enzymatic systems is to enhance the cleanliness of fibers [213,214]. Biological treatments, appreciated for their environmental benefits and energy efficiency, were explored by Angelini and colleagues for their potential in processing ramie fibers. They investigated the use of enzyme-based versus traditional chemical (NaOH) methods for defibrillation, focusing on two strains of Clostridium felsineum L known for their pectonolytic capabilities. When applying an alkaline approach, the fibers were treated with 2 % NaOH and then boiled for 2 h. This chemical method proved to be superior in removing hemicellulose and lignin compared to the enzymatic treatments. Nonetheless, the effectiveness of the two enzyme strains in lignin and hemicellulose removal was comparable, with no significant differences observed. Additionally, the study found no notable differences in the fibers' tensile properties, whether they were processed using the alkaline or enzyme-based methods. Studies have been carried out comparing the effects of enzymatic treatments and NaOH treatments on Jute fibers. These investigations have revealed that Xylanase enzyme treatment leads to the highest increase in Young's modulus, particularly when evaluating the impacts of different enzymatic mixtures and the duration of treatment. Furthermore, enzymatic treatments have been found to improve the fiber's aspect ratio. This enhancement is achieved through the breakdown of pectin, hemicellulose, and lignin, which are present on the surfaces of the lignocellulose fibers [[215], [216], [217]]. The research underscores the viability of enzyme treatments as a green and efficient approach to enhance the quality of fibers within the composite manufacturing sector.

## 6 Applications of bast fibers in the present century: a sustainable revolution

**Fig. 5:**
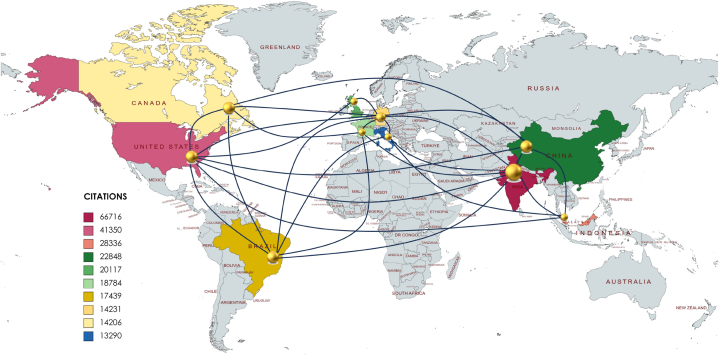
Main countries with their publication on man-made fibers, and natural fibers used in hybrid and polymeric composites from 2000 to 2022.

The top 10 countries have been instrumental in pushing the boundaries of research into natural fiber composites, showcasing a strong commitment to the sustainability and innovation objectives of the 2030 agenda. Through joint efforts, significant financial contributions, and well-planned policy measures, these nations are at the forefront of developments in natural fiber composites. These materials are pivotal for progress in environmentally friendly solutions and the discovery of new uses. India, the United States, Malaysia, China, the United Kingdom, France, Brazil, Germany, Canada, and Italy play a critical role in guiding research in this area, as illustrated in Fig. 5. Their collective actions, investment, and policies have established them as leaders in advancing this field. Incorporating examples from various engineering sectors enhance understanding of these advancements. For instance, in the automotive industry, natural fiber composites are being used to reduce vehicle weight significantly, thereby improving fuel efficiency and reducing carbon emissions. Studies have shown that parts made from these composites reduce weight by up to 30 %, contributing to a greener transportation sector [218]. In the construction industry, natural fiber composites are being applied in the fabrication of more sustainable building materials. These materials not only offer reduced environmental impact due to their biodegradability but also provide improved insulation properties, contributing to energy-efficient buildings. The lifespan of structures made with natural fiber composites be substantially longer due to the durability and corrosion resistance of these materials, which is a significant advantage over traditional construction materials. By focusing on these practical applications and their benefits, such as weight reduction in automotive parts and the extended lifespan of construction materials, the narrative becomes more engaging and illustrative of the tangible impacts of natural fiber composites in various engineering fields.

### 6.1 India

In the realm of high-impact research on natural fiber composites, India has emerged as a frontrunner, securing the top position with 3136 articles published and an impressive 66,716 citations. The significance of India's leadership in this field extends beyond sheer numbers, reflecting a strategic alignment with the global 2030 agenda for sustainable development. India's prowess in natural fiber composite research is rooted in its rich biodiversity and a long-standing tradition of utilizing natural resources. The country's scientists and researchers have harnessed this wealth to delve into the development of sustainable materials, particularly focusing on natural fibers. From exploring novel fiber sources to optimizing composite manufacturing processes, Indian researchers have demonstrated a comprehensive approach to advancing the field. By addressing the environmental impact of traditional materials through natural fiber composites, India actively contributes to the global pursuit of sustainable solutions. India boasts a robust research ecosystem, with premier academic institutions, research organizations, and dedicated research centers fostering innovation.

That way, in the realm of civil engineering, Indian researchers have delved into the development of sustainable building materials using natural fibers. From reinforced concrete structures to eco-friendly insulation materials, India's focus on natural fiber composites has contributed to environmentally conscious construction practices. For example, the use of jute fibers in concrete to enhance its tensile strength and durability, leading to more resilient and sustainable infrastructure.

Research initiatives have looked also utilizing fibers such as sisal and coir for manufacturing lightweight yet strong composite materials. In the realm of textile engineering, India's expertise in natural fibers has led to innovations in fabric composites. The use of fibers such as banana and pineapple for creating sustainable textiles with enhanced properties. These textiles find applications in clothing and home furnishings promoting a circular economy approach to textile production. Collaborations between academia and industry have further propelled the translation of research findings into practical applications. Government initiatives and funding support have incentivized researchers to explore sustainable alternatives, driving the growth in publications and citations. This collaborative approach enhances the quality and impact of research, placing India at the forefront of the global natural fiber composite research community. India's top position in high-impact research on natural fiber composites is a testament to its commitment to sustainability, aligned with the 2030 agenda.

### 6.2 United States

The United States has established itself as a leader in influential research on natural fiber composites, ranking second with 918 published articles and garnering 41,350 citations. This leadership reflects the country's dedication to scientific innovation and sustainability, aligning with the goals of the 2030 agenda. U.S. researchers and institutions have delved into the various aspects of natural fibers, contributing significantly to the discourse on sustainable materials. Such efforts support responsible production and climate goals, resulting from a strategic blend of academia, industry, and policy that fosters research excellence and innovation. As a result, the U.S. sets the pace in natural fiber composite research, paving the way for future sustainable advancements. This collaborative spirit has accelerated the pace of discovery and innovation, placing the U.S. at the forefront of this dynamic field. The investments have not only propelled the quantity of research but have also ensured a high impact, reflected in the substantial citation count. The policy framework has created an ecosystem where innovation thrives, leading to groundbreaking discoveries in natural fiber composites. The synergy between academia and industry in the United States has been a key driver of success in natural fiber composite research. As the world progresses towards a more sustainable future, the United States continues to lead the charge in pioneering research that will shape the landscape of natural fiber composites for years to come.

### 6.3 Malaysia

Malaysia ranks third globally in natural fiber composite research, with 1261 published articles and 28,336 citations, highlighting its dedication to scientific advancement in sustainable materials. The country leverages its rich local resources for developing natural fiber composites, aligning with sustainable development goals and driving industrial innovation. Through strategic investments in infrastructure, talent, and policy-making, Malaysia fosters a robust research environment. Partnerships between researchers and industry ensure practical applications for their findings, fostering economic growth and environmental sustainability. This strategic focus establishes Malaysia as a leading hub for natural fiber composite research and a contributor to global sustainability efforts. The Malaysian government has implemented strategic policies that prioritize research and development in sustainable materials, particularly natural fiber composites. Researchers actively engage with industrial partners, ensuring that their work addresses real-world challenges and contributes to the development of practical, market-ready solutions. Malaysia has invested in building the capacity of its research community by fostering talent development programs and educational initiatives. This emphasis on human capital ensures a sustainable pipeline of skilled professionals who contribute to the growth and continued excellence of natural fiber composite research. The emphasis on environmentally friendly materials positions Malaysia as a pioneer in promoting sustainable practices and mitigating the impact of traditional materials on the environment. Innovations born out of this research are driving economic growth and fostering a culture of innovation within the nation. The nation's leadership in this field not only positions Malaysia as a global research hub but also contributes significantly to the realization of sustainable development goals on a global scale.

### 6.4 China

China has rapidly become a prominent force in high-impact research on natural fiber composites, ranking 4th globally with 900 publications and over 22,000 citations. This surge reflects China's dedication to sustainable development, contributing to eco-friendly material science and supporting Sustainable Development Goals, particularly responsible consumption and increased climate action. Substantial investment in R&D and collaborative efforts between academia, industry, and international bodies have bolstered China's research capacity. Moreover, the government's push for sustainability has led to the creation of innovation-friendly environments. China's strategic focus on sustainable materials demonstrates its role in fostering a more resilient, eco-conscious future. The establishment of innovation hubs, research centers, and technology parks focused on sustainable materials has fostered an ecosystem conducive to breakthroughs in natural fiber composite research. China's commitment to environmental sustainability is reflected in its policy frameworks, encouraging industries to adopt green practices and invest in environmentally friendly materials, including natural fiber composites. China's impressive standing as the 4th leading country in high-impact natural fiber composite research attests to its strategic vision, concerted efforts, and substantial investments in advancing sustainable materials. China is not only contributing to global scientific knowledge but also actively participating in the global endeavor towards a more sustainable and resilient future.

### 6.5 United Kingdom

The UK stands as a leader in natural fiber composite research, ranking 5th globally with 470 publications and over 20,000 citations. This success reflects the UK's dedication to the development of eco-friendly composite materials, aligning with international sustainability goals like the 2030 Agenda for Sustainable Development. Backed by strong collaborations between academia, industry, and government, and supported by significant investments in infrastructure, UK researchers are making notable advances in the field. Policies promoting sustainable materials have spurred exploration into natural fiber composites, leading to their increased use in automotive, construction, and packaging. The UK's research initiatives not only contribute to the push for sustainability but also serve as a model for other nations' efforts in sustainable material innovation. As the world advances towards a more sustainable future, the United Kingdom's emphasis on high-impact research in natural fiber composites not only aligns with global goals but also sets an example for other nations. Through concerted efforts, strategic investments, and supportive policies, the UK continues to be a driving force in the evolution of composite materials, contributing significantly to the global discourse on sustainable and innovative solutions.

### 6.6 France

France has positioned itself as a global leader in natural fiber composite research, ranking 6th internationally with 543 publications and 18,784 citations. This reflects France's dedication to sustainable material development, supporting global research targets for 2030 and the Sustainable Development Goals (SDGs). French research impact is bolstered by collaborations among academia, industry, and the government, along with public and private funding. Policies encouraging eco-friendly materials have spurred innovative natural fiber applications, with international partnerships enhancing research diversity. France's active role in the sustainable materials sector exemplifies its commitment to shaping a sustainable future. The collaboration between research institutions and industries has facilitated the translation of scientific findings into practical applications, driving innovation and reinforcing France's global standing in the field. Partnerships with research institutions, organizations, and experts from around the world have enriched the diversity of perspectives and methodologies, contributing to the comprehensive understanding of natural fiber composites. Through international conferences, publications, and collaborative projects, France contributes to shaping the discourse and advancements in the field on a global scale. The nation's commitment to innovation and collaboration positions it as a driving force in shaping the future of sustainable materials.

### 6.7 Brazil

Brazil has claimed the 7th spot globally in high-impact research on natural fiber composites with 686 articles and 17,439 citations. This reflects the country's strategic focus and investment in this area, leveraging its rich biodiversity and abundant resources like jute, sisal, and banana fibers. Aligned with the UN's 2030 agenda for sustainability, Brazil's research contributes to eco-friendly initiatives and a circular economy. Collaborative efforts among academics, researchers, and the industry have spurred innovation, with investments in research infrastructure and supportive policies bolstering Brazil's role in developing sustainable solutions. Policy initiatives, including supportive frameworks and incentives for research and development, have created an enabling environment for scientists and industries to explore the full potential of natural fiber composites. Brazil's 7th place in global high-impact research on natural fiber composites is a testament to its proactive stance in addressing the challenges of the 21st century. This positioning not only strengthens Brazil's scientific standing but also positions the country as a key player in contributing to sustainable solutions for the world.

### 6.8 Germany

Germany holds the 8th rank globally in impactful research, with 488 published articles and 14,231 citations, reflecting its strong commitment to scientific advancement, particularly in natural fiber composite research. This achievement aligns with the global sustainability goals set for 2030. The country's research thrives on a collaborative ethos involving academia, research centers, and industry, which propels innovation and practical applications. Generous R&D funding supports the exploration of sustainable technologies, aligning with policies that favor green technology and the circular economy. German research contributes to the 2030 sustainability agenda, with a special focus on eco-friendly composites. This international cooperation and commitment to sustainability highlight Germany's influential role in shaping the future of natural fiber composite research.Natural fiber composites, known for their sustainability and reduced carbon footprint, have emerged as a key focus area in response to the imperative for eco-friendly solutions. This openness to global collaboration enhances the impact of its research and contributes to the cross-pollination of ideas in the field of natural fiber composites. The convergence of concerted research efforts, substantial investments, strategic policy initiatives, and alignment with the 2030 agenda has catapulted Germany into a leading position, shaping the future of natural fiber composite research on both national and global scales.

### 6.9 Canada

Canada has emerged as a leading force in natural fiber composite research, ranking 9th globally with 418 published articles and over 14,000 citations. The country's research, focusing on materials like flax, hemp, and wood fibers, contributes to the global pursuit of sustainable alternatives to synthetic materials. This commitment supports the 2030 Agenda for Sustainable Development, with particular regard to responsible production and consumption. Through strategic investments and collaborations among academia, industry, and government, Canada has not only advanced its scientific standing but also spurred economic growth in the sustainable materials sector. This holistic approach has not only propelled Canada's standing in global research but has also stimulated economic growth and job creation within the burgeoning field of sustainable materials. Canada's remarkable position as a top contributor to high-impact research in natural fiber composites reflects its commitment to sustainability, alignment with the 2030 Agenda, and a strategic approach involving concerted efforts, investments, and supportive policies.

### 6.10 Italy

Securing 10th in global high-impact research with 379 publications and 13,290 citations, Italy is at the vanguard of natural fiber composite research, aligning with the UN's 2030 sustainable development goals. Italy's strategy leverages natural fibers for innovative, eco-friendly materials. Investment in research infrastructure and collaborations between academia and industry has advanced its market position. Policies fostering sustainability, alongside specialized education, bolster Italy's role in sustainable material innovation, positioning it as a pivotal contributor to a resilient future. Specialized courses, research fellowships, and collaborative projects provide students and researchers with the knowledge and skills needed to contribute to the advancement of this critical field. By prioritizing real-world impact, Italy has positioned itself as a key player in the global market for sustainable and high-performance materials. A dynamic research landscape, responsive to evolving challenges and opportunities, ensures that Italy remains at the forefront of innovation in this critical area. As Italy continues to lead in this field, its contributions are expected to play a pivotal role in shaping a sustainable and resilient future.

**Fig. 6:**
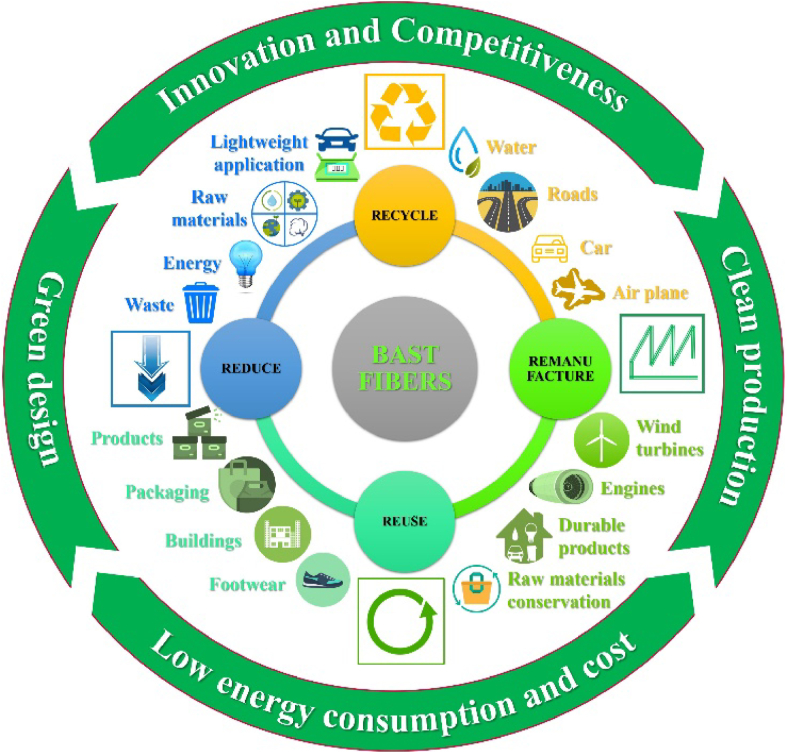
Circular economy cycle model applied to bast fibers.

In the 21st century, bast fibers have found an increasingly prominent role in a variety of applications across industries, owing to their remarkable environmental benefits and the pursuit of sustainability. This section delves into the contemporary and burgeoning applications of bast fibers, underscoring the pivotal role they play in reducing the carbon footprint, lowering energy consumption, and decreasing dependence on petrochemical-based materials. Additionally, we address the challenges faced in their adoption and the exciting future prospects for this eco-friendly alternative. Bast fibers are defined as renewable materials. These materials boast mechanical properties, economic viability, biodegradability, renewability, and low density. Emerging sustainable fiber composites find application in components or products where the applied load intensity isn't excessively high [194]. Particularly, in applications where low mechanical performance is sufficient, the use of bast fibers proves pivotal, contributing to pollution reduction [195,196]. Leveraging widely available materials paves the way for the development of cost-effective composites capable of efficiently replacing conventional, relatively expensive materials [197]. Sustainable fiber reinforcement composites centered around bast fibers demonstrate significant potential for development in the realm of ecological, lightweight, and sustainable composites [198]. The pathway forward involves the identification of new bast fibers, their residues, their economically and environmentally sound production, and the implementation of interfacial properties achieved through chemical/physical treatments in fibers [192]. Bast fibers exhibit several compelling properties over man-made fibers, including biodegradability, low cost, specific stiffness (E/ρ), easy availability, and reduced weight, affording bast fiber composites a superior standing in unique applications compared to man-made fiber-reinforced composites [199]. While technical man-made fibers are commonly employed in advanced structural materials [200], a transformative shift has occurred in these applications, with sustainable materials taking the forefront due to the depletion of inorganic materials derived from petroleum and other mineral sources [201]. These sustainable materials have garnered significant attention as environmentally friendly alternatives to petrochemical fibers in new engineering composites [202]. Bast fibers present a range of technological and ecological advantages aligning with conceptual design strategies, as depicted in Fig. 6 [203]. Despite several ecological advantages over petrochemical and mineral fibers, bast fibers are currently emerging as promising alternatives within conceptual design strategies, contributing to the mechanized growth of new sustainable materials and products in comparison to other fibers [204,205].

The clamor for sustainability has changed the game in the fields of applications of composites: Towards Conceptual design strategies.

### 6.11 Requirements of new materials and products

I. Renewability, recyclability, biodegradability, abundant, cheap.
II. Lower environmental impacts.
III. Improves fuel efficiency and reduces emissions.
IV. End of life incineration of bast fibers results in energy and neutral CO_2_.
V. Good mechanical acoustic and thermal properties.
VI. low weight, low density, and low cost.
VII. No skin irritations.

Innovations in processing and technology are expected to revolutionize bast fiber applications across industries. Bast fibers are experiencing a renaissance in the 21st century, offering sustainable and renewable alternatives to man-made fibers. Their environmental benefits, from reduced carbon footprint to lower energy consumption, are at the forefront of this transformation. While challenges persist, ongoing research and innovation are propelling bast fibers into a promising future, where they can significantly contribute to the sustainability and renewable resource utilization goals of various industries.

## 7 Conclusions, future prospects, and global trend for bast fibers

The exploration and utilization of bast fibers have witnessed significant strides, marking a pivotal shift towards sustainable and versatile materials in various industries. The inherent strength, lightweight nature, and eco-friendly characteristics of bast fibers position them as attractive alternatives to conventional materials. The existing applications in packaging, aerospace, building, and automotive sectors underscore their potential to address the demands of a rapidly evolving market that prioritizes environmental sustainability. The future of bast fibers holds promising avenues for growth and innovation. One crucial aspect is the ongoing research aimed at improving the chemical structures of bast fibers, enhancing their mechanical properties, and expanding their range of applications. Innovations in fiber processing techniques and chemical modifications could lead to the development of fibers with superior strength, increased flexibility, and enhanced resistance to environmental factors. Another key area of future exploration lies in expanding the applicability of bast fibers. As technologies advance, there is potential to discover new applications in sectors yet to fully harness the benefits of these fibers. The integration of bast fibers in unknown or underutilized manufacturing sectors could unlock novel possibilities, creating a ripple effect in industries seeking sustainable alternatives. A global trend towards sustainability and environmental consciousness is propelling the demand for bast fibers. As consumers and industries alike prioritize eco-friendly practices, bast fibers are poised to play a crucial role in meeting these expectations. Governments and regulatory bodies are increasingly advocating for sustainable materials, and this trend is likely to drive research and investment in bast fiber technologies on a global scale. The adoption of bast fibers aligns with the overarching trend of circular economies, where materials are sourced responsibly, utilized efficiently, and disposed of sustainably. This shift towards a more circular approach is reshaping industries, and bast fibers are well-positioned to contribute significantly to these transformative changes. In conclusion, the future of bast fibers is bright, with ongoing research and development efforts expected to further enhance their properties and broaden their applications. As global awareness of environmental issues grows, the demand for sustainable alternatives like bast fibers will likely continue to rise, contributing to a more sustainable and resilient future for multiple industries.

## CRediT authorship contribution statement

**Caroliny M. Santos:** Writing – review & editing, Writing – original draft, Validation, Methodology, Investigation, Formal analysis, Data curation, Conceptualization. **Thiago F. Santos:** Visualization, Methodology, Investigation, Formal analysis, Data curation, Conceptualization, Writing – original draft, Writing – review & editing. **Marcos S. Aquino:** Writing – review & editing, Visualization, Validation, Supervision, Project administration, Methodology, Investigation, Funding acquisition, Formal analysis, Data curation, Conceptualization. **Sanjay Mavinkere Rangappa:** Methodology, Investigation, Formal analysis, Data curation, Conceptualization, Supervision, Validation, Visualization, Writing – original draft, Writing – review & editing. **Suchart Siengchin:** Writing – review & editing, Supervision, Project administration, Methodology, Funding acquisition, Conceptualization. **Indran Suyambulingam:** Writing – review & editing, Validation, Methodology, Investigation, Conceptualization.

## Declaration of competing interest

The authors declare that they have no known competing financial interests or personal relationships that could have appeared to influence the work reported in this paper.
